# The prevalence of developmental defects of enamel in people with cystic fibrosis: a systematic review

**DOI:** 10.1186/s12903-024-04227-4

**Published:** 2024-04-12

**Authors:** Fiona O’Leary, Niamh Coffey, Martina Hayes, Francis Burke, Mairéad Harding, Barry Plant

**Affiliations:** 1 Cork University Dental School & Hospital, Wilton, Cork Ireland; 2 University College Cork, Wilton, Cork Ireland; 3 Dublin Dental University Hospital, Trinity College Dublin, Dublin, Ireland; 4 University College Cork, Cork, Ireland

**Keywords:** Cystic fibrosis, Developmental Defects of Enamel, Oral health, Public Health Policy, Policy Development, Prevention.

## Abstract

**Background:**

Oral health impacts systemic health, individual well-being, and quality of life. It is important to identify conditions that may exacerbate oral disease to aid public health and policy development and promote targeted patient treatment strategies. Developmental defects can increase an individual’s risk of dental caries, hypersensitivity, premature tooth wear, erosion, and poor aesthetics. As part of an ongoing study assessing oral health in adults with cystic fibrosis at Cork University Dental School and Hospital, a systematic review of available literature was conducted to assess the prevalence of enamel defects in people with cystic fibrosis.

**Aims:**

To critically evaluate the literature to determine if the prevalence of developmental defects of enamel is higher in people with cystic fibrosis (PwCF).

**Methods:**

Data Sources: Three online databases were searched Embase, Scopus, and Web of Science Core Collection. Studies that examined an association between cystic fibrosis and developmental defects of enamel were included in this systematic review.

**Results:**

The initial search identified 116 publications from the following databases Embase, Web of Science Core Collection, and Scopus. Eleven studies were included for qualitative analysis. Nine studies concluded that PwCF had a higher prevalence of enamel defects than control people and one study found no difference in cystic fibrosis (CF) status. All studies had a risk of bias that may influence study results and their interpretation.

**Conclusions:**

The results of the systematic review show a consistent pattern that PwCF have a higher prevalence of DDE than people without CF. Genetic dysfunction, chronic systemic infections, and long-term antibiotic use are possible aetiological causes. This review highlights the need for future studies to investigate if DDEs are caused by the underlying CFTR mutation or as a consequence of disease manifestations and/or management.

**Supplementary Information:**

The online version contains supplementary material available at 10.1186/s12903-024-04227-4.

## Introduction

Developmental defects of enamel (DDE) are disturbances in the quality and/or quantity of enamel which present as enamel hypoplasia and/or enamel hypomineralisation. Enamel hypoplasia is a quantitative defect of enamel presenting clinically as surface pits and groves, enamel absence, and microdontia. Enamel hypomineralisation is a qualitative defect of mineral content resulting in the discolouration of teeth. Enamel defects may affect a single tooth or multiple or groups of teeth [1]. Individuals with DDE may be at a greater risk of oral pathologies such as dental caries, hypersensitivity, and premature tooth wear [2]. DDE also poses a considerable aesthetic concern for patients [3]. Additional dental concerns associated with the presence of DDEs include dental behavioural problems such as dental fear and anxiety [1].

DDEs are caused by diverse interacting genetic and environmental factors. There is a higher prevalence of DDEs in individuals with genetic conditions such as amelogenesis imperfecta and ectodermal dysplasia [4] and systemic conditions such as coeliac disease. People with cystic fibrosis (CF) have also been identified as having a higher prevalence of enamel defects. CF is an autosomal recessive condition caused by a mutation of the cystic fibrosis transmembrane conductance regulator gene (CFTR). Pulmonary manifestations of the disease are most widely recognised; however, multiple organ systems are affected by the condition. The exact aetiology of DDEs within this population is not fully understood, reports from animal studies indicate that the dysfunctional CFTR gene may be responsible. Other possible aetiologies include recurrent systemic infection, long- term antibiotic use, and nutritional malabsorption similar to that seen in coeliac disease.

Oral health in CF populations is under-researched possibly as a consequence of historic premature mortality, however, people with cystic fibrosis (PwCF) are now living longer. This is a consequence of multifaceted developments in the management of the disease particularly the introduction of gene modulator therapies. These target the underlying genetic mutation and were introduced in 2012. Previous studies have identified the oral cavity as a reservoir for bacteria capable of colonising the pulmonary system [5]. It is imperative to identify if people with this condition are at a greater risk of developing oral disease so that preventative programmes and targeted patient care can be developed. These are some of the catalysts for conducting research into the oral health in the CF population. There are currently no guidelines for the provision of dental care for PwCF. Therefore, it is timely to review the existing evidence regarding oral manifestations of CF specifically enamel defects. This can identify gaps in research and aid in future research design. The objective of this systematic review was to identify if PwCF have a higher prevalence of DDE than people without CF. A systematic review of the current literature was conducted. The following research question was formulated using the PICO framework; Is the prevalence of developmental defects of enamel higher in people with cystic fibrosis compared to people without cystic fibrosis?

## Materials and methods

### Eligibility criteria

Studies were screened according to the following inclusion and exclusion criteria. The inclusion criteria were formed based on the parameters of the review PICO (study population: people with cystic fibrosis, intervention: developmental defects of enamel). Articles published between 1960 and 2023 permitted a larger search scale and allowed the authors to determine if any time trends in the prevalence of DDEs existed. Only primary literature sources were used, hence review articles were excluded.

### Inclusion criteria

- Studies involving human subjects.
- Studies that were of cohort, cross-sectional or case-control design.
- Study subjects had a positive diagnosis of cystic fibrosis.
- Studies published from 1960 to 2023.
- Developmental defects of enamel reported for deciduous and/or permanent teeth.

### Exclusion criteria

- Studies involving animal subjects.
- Review articles.

### Information sources

The searches were conducted in the following electronic databases: Embase, Web of Science Core Collection, and Scopus. The bibliographies of relevant publications were manually screened to identify additional publications not found in the electronic databases.

### Search strategy and selection criteria

The following search terms “cystic fibrosis” AND “enamel defect”, “cystic fibrosis” AND “dental hard tissue”, “cystic fibrosis” AND “developmental enamel defect”, “cystic fibrosis” AND “dental hypoplasia”, “cystic fibrosis” AND “dental hypomineralisation” were used. Identical methods were used to search for all relevant publications in all three databases. The final search was conducted on the 13th of December 2023. An example of a search strategy is included in Appendix 1.

Two investigators (FO’L & NC) screened titles and abstracts to determine eligibility. Full articles were obtained for identified titles and those that met eligibility were included in the review. Investigators (FO’L & NC) worked independently, and any differences in opinion were reviewed by a third investigator (MH).

### Data items

The following data were extracted by two investigators (FO’L & NC) from studies meeting the eligibility criteria: year of study, type of study, participant ages, sample size, prevalence of enamel defects, statistical methods, and study conclusions.

## Results

### Study selection

**Fig. 1:**
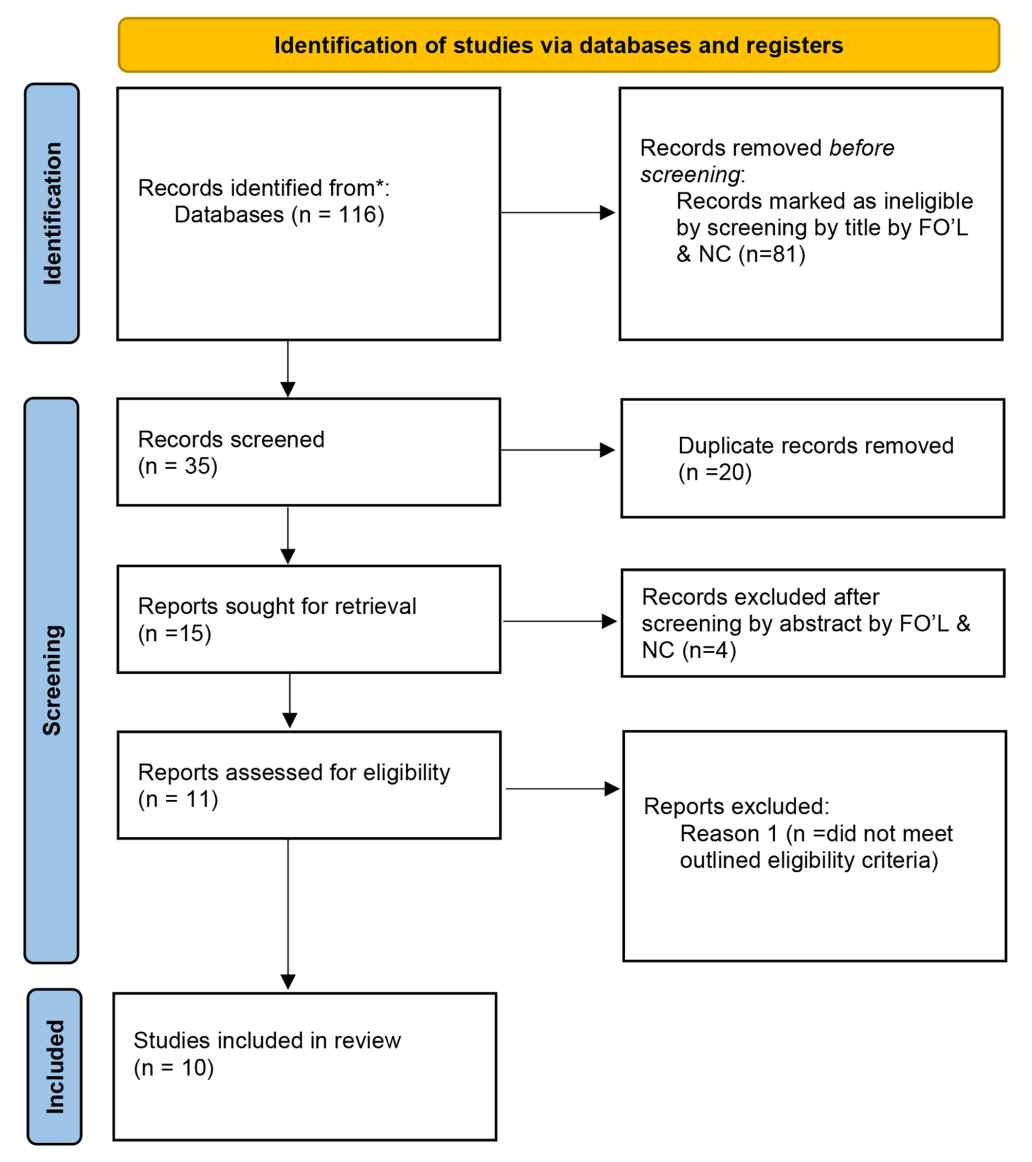
Flow chart indicating the number of records identified and included for qualitative analysis on developmental defects of enamel in people with cystic fibrosis

The search resulted in 116 publications from Embase, Web of Science Core Collection, and Scopus. Following title and abstract screening, 11 publications were included for full-text screening. Two publications referred to the same study so were therefore considered as one. Ten studies were included for qualitative analysis (Fig. 1).

### Study characteristics

Of the ten studies included, three were conducted in the USA [6–8], five in Europe [9–13], one in Brazil [14],and one in Turkey [15]. A total of 476 people with CF were studied. The earliest study included was published in 1976^6^, with the most recent study published in 2019^12^.

Eight studies included children and adolescents with CF up to the age of eighteen^6,8–11,13−15^. One study included both children and adults with CF over the age of eighteen [7]. One study limited study participants to adults with CF over the age of eighteen [12].

Several different clinical indices were used to categorize enamel defects. Four studies used the modified developmental defects of enamel (mDDE) index [8, 10–12], and one study used the DDE index [14]. The study conducted by Narang [9] categorized enamel defects based on WHO criteria. Primosch [7] measured defects using criteria developed by Russel [16], whereas Peker used criteria developed by Weerheijm [17]. The study conducted by Jagels and Sweeney [6] measured enamel defects as a percentage of teeth showing hypomineralisation.

Eight studies included control groups [6, 7, 9–12, 14, 15], and only two studies did not [8, 13]. Six studies included age and gender-matched ‘healthy’ individuals in the control group [7, 10–12, 14, 15], one study included non-CF siblings in the control group [6], and another study included control subjects with chronic respiratory conditions [9]. This data is summarised in Table 1.

**Table 1:** Tabulated Results showing the Author, Year of Study, Number of Individuals Examined, Age Group, and Enamel Defects Measurement

Author	Type of Study	Study Population	Age Group(years)	DDE Measurement
Jagels&Sweeney 1976	Case-control study	Case 63 Control 56	5–12	% of subjects showing enamel hypoplasia
Primosch 1980	Cross-sectional observational study	Case 86 Control 86	3.3–24.2	% of DDE in permanent teeth
Narang 2003	Case-control study	Case 74 Control 106	Case 2.5–16.5 Control 3.0– 16.5	% of opacities in primary teeth % of opacities in permanent teeth
Azevedo 2006	Case-control study	Case 13 Control 13	Mean age 10.3	% of permanent teeth affected according to DDE type
Ferrazzano 2009	Case-control study	Case 54 Control 101	7–12	% of subjects with DDE
Ferrazzano 2012	Case-control study	Case 88 Control 101	4–12	Prevalence of DDE present (%)
Peker 2014	Case-control study	Case 30 Control 30	Case 10.2 ± 4.8 Control 9.9 ± 1.4	% of subjects with mild DDE, moderate DDE, severe DDE
Collard 2016	Cross-sectional observational study	Case 26 Control N/A	< 18	% of subjects with hypomineralised teeth
Abu-Zahra 2019	Cross-sectional study	Case 20 Control N/A	6–18	% of subjects with DDE
Pawlaczyk-Kamieńska 2019	Cross-sectional study	Case 22 Control 22	20–43	% of subjects with DDE

Four studies reported a statistically significant increase in the prevalence of DDE in PwCF compared to non-CF control groups [8, 10–12]. The study by Narang [9] reported a statistically significant difference in the prevalence of DDE in the CF control group aged 6–9 years only. Two studies reported a non-significant increase in the prevalence of DDE between CF a non-CF control groups [14, 15]. Primosch [7] found that DDE were more common in people with CF compared to healthy individuals. Two studies by Jagels and Sweeney [6] and Collard [13] reported no difference in the prevalence of enamel defects between PwCF and non-CF people/ national averages. These results are summarised in Table 2.

**Table 2:** Data abstracted from studies regarding developmental defects of enamel in People with Cystic Fibrosis

Author	DDE Status	Conclusion
Jagels&Sweeney 1976	Case 5% Control 1%	There is no significant difference in the percentage of subjects with DDE between the case and control groups.
Primosch 1980	Case 33% Control 13–15%	There was a higher incidence of DDE in the case group when compared to the control group.
Narang 2003	Case 10% (primary teeth) 41% (permanent teeth) Control 11% (primary teeth) 21% (permanent teeth)	Higher % of opacities in incisor and molar teeth in the case group compared to the control group.
Azevedo 2006	Case 39% (demarcated opacity) 15% (diffuse opacity) 3% (hypoplasia) Control 11% (demarcated opacity) 17% (diffuse opacity) 2% (hypoplasia)	Higher prevalence of demarcated and hypoplastic DDE in the case group compared to the control group.
Ferrazzano 2009	Case 55.6% Control 22%	A statistically significant higher prevalence of DDE in the case group.
Ferrazzano 2012	Case 33% (primary teeth) 56% (permanent teeth) Control 20% (primary teeth) 23% (permanent teeth)	There is a higher prevalence of DDE in the case group.
Peker 2014	Case 83.3% (mild) 16.7% (moderate) 0% (severe) Control 100% (mild) 0% (moderate) 0% (severe)	Higher prevalence of DDE in the case group.
Collard 2016	Case 15%	A similar proportion of enamel defects in the case group when compared to the national Welsh average.
Abu-Zahra 2019	Case 50% (permanent teeth) 60% (first permanent molars and Incisors)	Higher prevalence of DDE in the case group compared to national averages.
Pawlaczyk-Kamieńska 2019	Case 54.55% Control 22.73%	Higher prevalence of DDE in the case group.

### Risk of Bias

The risk of bias was assessed using several headings. These were adapted from previous reviews in this subject field by Chi et al. [18] and Coffey et al. [19]. This assessment was conducted by two investigators working independently (FO’L & NC). Any conflict was reviewed by a third investigator (MH). Studies meeting all seven criteria were classified as being at low risk of bias. Studies meeting three to five criteria were classified as medium risk of bias. Studies meeting less than three criteria were classified as being at high risk of bias. Seven studies were classified as being at medium risk of bias, and three as a high risk [6, 7, 13]. Eight studies included a control group [6, 7, 9–12, 14, 15]. Only one study provided an explanation for the study group [6]. The study by Jagels and Sweeney included non-CF siblings [6] in the control group. The study by Narang included individuals with chronic respiratory illness as control subjects [9]. The inclusion of such control groups may influence results as siblings may be carriers of the CFTR mutation, and people with respiratory illness may have a similar therapeutic history to PwCF. A limitation common to all studies was the absence of examiner blinding. The authors appreciate this may be difficult to achieve owing to the complex medical history of PwCF. Five studies were conducted by one examiner and did not provide sufficient information regarding examiner reliability [6, 7, 9, 14, 15]. Seven studies adopted standard defect measures (e.g., DDE, mDDE) [8–11, 13, 14], while three studies did not (Table 3) [6, 7, 15].

**Table 3:** Risk of bias assessment

Author	Inclusion of a control group	Justified selection of control group	Validated statistical analysis	Validated DDE measure	Examiner number	Examiners) blinded	Inter/ Intra examiner reliability	Risk of Bias
Jagels& Sweeney 1976	Yes	Yes, but controls were siblings of individuals with CF and may have been carriers of the CFTR gene	No	No	1	Yes, but case subjects were identifiable	Unknown	High
Primosch 1980	Yes	Yes	No	No	Unknown	Unknown	Unknown	High
Narang 2003	Yes	Yes, but the control group contained individuals with respiratory conditions	Yes	Yes	1	Unknown	Unknown	Medium
Azevedo 2006	Yes	Yes	No	Yes	1	Unknown	Unknown	Medium
Ferrazzano 2009	Yes	Yes	No	Yes	2	Unknown	Unknown	Medium
Ferrazzano 2012	Yes	Yes	No	Yes	3	Unknown	Yes	Medium
Peker 2014	Yes	Yes	Yes	No	1	No	Yes	Medium
Collard 2016	No	N/A	No	Yes	1	No	Unknown	High
Abu-Zahra 2019	No	N/A	Yes	Yes	2	No	Yes	Medium
Pawlaczyk-Kamieńska 2019	Yes	Yes	Yes	Yes	2	No	Yes	Medium

## Discussion

The objective of this review was to identify if PwCF had a higher prevalence of enamel defects compared to people without. The results of the review indicate that while there is not a unanimous agreement across all studies, there is a consistent pattern that the prevalence of enamel defects is higher in PwCF. Eight studies reported an increased prevalence of enamel defects in CF groups compared to non-CF control groups. Five of these studies reported a statistically significant increase [8–12], although the study by Narang et al. found this difference was limited to children aged between 6 and 9 years only [9]. The differences in enamel defect status in the additional three studies were not statistically significant [7, 14, 15]. Two studies reported no difference in the prevalence of enamel defects between groups [6, 13]. One of these studies by Jagels & Sweeney used siblings of individuals with CF as control participants. These individuals may carrier one of the CFTR mutations which may influence results. Interpretation of the study outcomes should be done with caution as all studies reviewed had design and/or methodological limitations that placed them at a medium or high risk of bias. The risk of bias and small sample sizes may reduce the validity and reliability of the study findings.

There are several limitations of the data included in this review. Studies used different measurement indices for enamel defects. Some studies measured defects at an individual level (percentage of individuals with enamel defects), while other studies measured at a tooth level (percentage of teeth with enamel defects). The absence of a standardised measurement criteria makes the comparability of studies challenging. Six studies were conducted by a single examiner. Therefore, we cannot exclude the possibility of examiner bias in these studies. Four studies [8, 11, 12] were conducted by multiple examiners which is a study strength however, only three provided information regarding examiner training and reliability. For ease of reproducibility and comparability, future studies should include details of examiners training and calibration using a WHO-approved or standardised measurement index.

Another limitation common to all studies was the recruitment of small sample sizes, with children and adolescents accounting for the majority of study participants. To increase study recruitment and sample sizes, future studies should use a multicentre approach. The majority of studies were conducted before the introduction of gene modulators such as Kaftrio® and Orizaba® in 2012 and can now be deemed historic. Only three studies [8, 15, 20] were conducted after the introduction of these medications. Gene modulators improve CFTR function, reduce pulmonary exacerbations and systemic manifestations of the disease [21]. However, PwCF included in these three studies would not have had the benefit of gene modulators during tooth development since mineralisation of permanent teeth occurs during the first three years of life. Children as young as four months old are now being offered gene modulator therapy. DDE in PwCF have been attributed to metabolic disturbances, long-term antibiotic, surgical intervention, and pancreatic enzyme use [11, 7, 8]. It is therefore reasonable to assume that with fewer disease manifestations and exacerbations requiring antibiotics, and surgical intervention as a result of improved CFTR function there may be a reduction in the number of enamel defects in children with CF.

This review was conducted to identify gaps in the area of oral health in PwCF. A strength was a clearly defined and reproducible search criteria which was conducted by two investigators independently (FO’L & NC). The inclusion of two investigators aims to reduce the risk of reviewer bias. The investigators included articles published between 1960 and 2023, publications were not confined to the English language which permitted a larger search scale. While the authors manually screened bibliographies there is a possibility that not all eligible publications were included which is a limitation of the study.

## Conclusion

The objective of the systematic review was met. A general trend that PwCF have more enamel defects than people without was identified. While the strength of the evidence to support this was moderate at best, if one considers the concepts of biological plausibility, accepted medical knowledge, and consistent result patterns the strength of the evidence is greatly improved. The presence of DDEs increases the risk of preventable oral diseases such as dental caries and erosion occurring, both can negatively impact an individual’s quality of life and masticatory function. Therefore, dental practitioners should be mindful of this so that early identification and appropriate treatment can be provided to the patient. Prevention and early intervention may afford PwCF the opportunity and tools to maintain their dentition into old age. The review has highlighted a need for future longitudinal studies taking a multi-centre approach assessing the effect that gene modulator therapies have on the incidence of DDE.

### Electronic supplementary material

Below is the link to the electronic supplementary material.

Supplementary Material 1

## Data Availability

The datasets used and/or analysed during the current study are included within this paper and available from online database Embase, Scopus, Web of Science Core Collection.

## References

[1] Jälevik B, Szigyarto-Matei A, Robertson A (2018). The prevalence of developmental defects of enamel, a prospective cohort study of adolescents in western Sweden: a barn I TAnadvarden (BITA, children in dental care) study. Eur Archives Pediatr Dentistry.

[2] Robles MJ, Ruiz M, Bravo-Perez M, González E, Peñalver MA. Prevalence of enamel defects in primary and permanent teeth in a group of schoolchildren from Granada (Spain). *Med Oral Patol Oral Cir Bucal*. Mar 1. 2013;18(2):e187-93. 10.4317/medoral.18580. 10.4317/medoral.18580 PMC3613868 23229271

[3] Sadana G, Gupta T, Rai HK (2019). Effect of esthetic defects in Anterior Teeth on the emotional and Social Well-being of children: a Survey. Int J Clin Pediatr Dentistry.

[4] Seow WK (2014). Developmental defects of enamel and dentine: challenges for basic science research and clinical management. Aust Dent J.

[5] Gomes-Filho IS, Passos JS, Da Cruz SS. Respiratory disease and the role of oral bacteria. *Journal of Oral Microbiology*. 2010;2(2010)10.3402/jom.v2i0.5811. 10.3402/jom.v2i0.5811 PMC3084574 21523216

[6] Jagels AE, Sweeney EA (1976). Oral health of patients with cystic fibrosis and their siblings. J Dent Res.

[7] Primosch RE. Tetracycline discoloration, enamel defects, and dental caries in patients with cystic fibrosis. *Oral Surgery, Oral Medicine, Oral Pathology*. 1980/10/01/1980;50(4):301–308. 10.1016/0030-4220(80)90411-9. 10.1016/0030-4220(80)90411-9 6935580

[8] Abu-Zahra R, Antos NJ, Kump T, Angelopoulou MV (2019). Oral health of cystic fibrosis patients at a north American center: a pilot study. Med Oral Patol Oral Cir Bucal May.

[9] Narang A, Maguire A, Nunn JH, Bush A (2003). Oral health and related factors in cystic fibrosis and other chronic respiratory disorders. Arch Dis Child.

[10] Ferrazzano GF, Orlando S, Sangianantoni G, Cantile T, Ingenito A (2009). Dental and periodontal health status in children affected by cystic fibrosis in a southern Italian region. Eur J Paediatr Dent Jun.

[11] Ferrazzano GF, Sangianantoni G, Cantile T, Amato I, Orlando S, Ingenito A (2012). Dental enamel defects in Italian children with cystic fibrosis: an observational study. Community Dent Health.

[12] Pawlaczyk-Kamieńska T, Borysewicz-Lewicka M, Śniatała R, Batura-Gabryel H. Clinical evaluation of the dental hard tissues in an adult population with cystic fibrosis. Pol Archives Intern Med. 2019. 10.20452/pamw.14918 31379359

[13] Collard M, Azzopardi KA, Hall SJ, Jones V, Thia LP. Oral health in children with cystic fibrosis. Conference Abstract. *Journal of Cystic Fibrosis*. 2016;15:S87-S88.

[14] Azevedo TDPL, Feijó GCS, Bezerra ACB. Presence of developmental defects of enamel in cystic fibrosis patients. J Dentistry Child (Chicago Ill). 73(3):159–63. 17367033

[15] Peker S, Mete S, Gokdemir Y, Karadag B, Kargul B (2014). Related factors of dental caries and molar incisor hypomineralisation in a group of children with cystic fibrosis. Eur Archives Pediatr Dentistry.

[16] THE DIFFERENTIAL DIAGNOSIS OF FLUORIDE, RUSSELL AL, AND NONFLUORIDE ENAMEL OPACITIES (1961). J Public Health Dent.

[17] Weerheijm KL, Mejàre I (2003). Molar incisor hypomineralization: a questionnaire inventory of its occurrence in member countries of the European Academy of Paediatric Dentistry (EAPD). Int J Paediatr Dent Nov.

[18] Chi DL (2013). Dental caries prevalence in children and adolescents with cystic fibrosis: a qualitative systematic review and recommendations for future research. Int J Paediatr Dent Sep.

[19] Coffey N, O’ Leary F, Burke F, Roberts A, Hayes M. Periodontal and oral health status of people with Cystic Fibrosis: a systematic review. *Journal of dentistry*. 2020 12 (Epub 2020 Oct 2020;103:103509. 10.1016/j.jdent.2020.103509. 10.1016/j.jdent.2020.103509 33129998

[20] Pawlaczyk–Kamieńska T, Borysewicz–Lewicka M, Śniatała R, Batura–Gabryel H (2019). Clinical evaluation of dental hard tissues in an adult population with cystic fibrosis. Article. Pol Archives Intern Med.

[21] Martin C, Guzior DV, Gonzalez CT et al. Longitudinal microbial and molecular dynamics in the cystic fibrosis lung after Elexacaftor–Tezacaftor–Ivacaftor therapy. *Respir Res*. 2023/12/16 2023;24(1):317. 10.1186/s12931-023-02630-z. 10.1186/s12931-023-02630-z PMC10725582 38104128

