# Supplementary material for: The prevalence of developmental defects of enamel in people with cystic fibrosis: a systematic review
Source: BMC Oral Health. 2024 Apr 12;24:446. doi: 10.1186/s12903-024-04227-4 (PMC11015619; doi:10.1186/s12903-024-04227-4)
Supplement: Supplementary file 1 — Supplementary Material 1 [file 12903_2024_4227_MOESM1_ESM.docx]

**Appendix 1**

Embase session results (26 Jun 2023)

No. Query Results

#7 ('cystic fibrosis'/exp OR 'cystic fibrosis') AND 'dental hypomineralisation' AND [<1966-2023]/py 0

#6 ('cystic fibrosis'/exp OR 'cystic fibrosis') AND 'dental hypoplasia' AND [<1966-2023]/py 1

#5 ('cystic fibrosis'/exp OR 'cystic fibrosis') AND 'developmental enamel defect' AND [<1966-2023]/py 0

#4 ('cystic fibrosis'/exp OR 'cystic fibrosis') AND ('dental hard tissue'/exp OR 'dental hard tissue') AND [<1966-2023]/py AND [english]/lim 38

#3 ('cystic fibrosis'/exp OR 'cystic fibrosis') AND ('dental hard tissue'/exp OR 'dental hard tissue') AND [<1966-2023]/py 38

#2 ('cystic fibrosis'/exp OR 'cystic fibrosis') AND ('enamel defect'/exp OR 'enamel defect') AND [<1966-2023]/py AND [english]/lim 5

#1 ('cystic fibrosis'/exp OR 'cystic fibrosis') AND ('enamel defect'/exp OR 'enamel defect') AND [<1966-2023]/py
